# ORF virus causes tumor-promoting inflammation in sheep and goats

**DOI:** 10.1177/03009858241241794

**Published:** 2024-04-13

**Authors:** Davide Pintus, Maria G. Cancedda, Giantonella Puggioni, Rosario Scivoli, Angela M. Rocchigiani, Caterina Maestrale, Elisabetta Coradduzza, Roberto Bechere, Luciana Silva-Flannery, Hannah A. Bullock, Simona Macciocu, Maria A. Montesu, Vincenzo Marras, Simone Dore, Jana M. Ritter, Ciriaco Ligios

**Affiliations:** 1 Istituto Zooprofilattico Sperimentale della Sardegna, Sassari, Italy; 2 Centers for Disease Control and Prevention, Atlanta, GA; 3 Università degli studi di Sassari, Sassari, Italy

**Keywords:** CD163+ macrophages, epidermal growth factor receptor 2, gene expression, goat, parapoxvirus, sheep, tumor, vascular endothelial growth factor

## Abstract

ORF virus (ORFV) causes contagious ecthyma (“ORF”), a disease of sheep and goats characterized by lesions ranging from vesicles and pustules to atypical papilloma-like and angiomatous lesions in the skin and mucosae. The authors investigated the molecular factors leading to the ORF-associated atypical tumor-like changes. Fifteen lambs, 15 kids, and an adult ram clinically affected by natural ORFV infection were enrolled in the study and examined by several methods. ORFV was detected by viral culture or real-time polymerase chain reaction (RT-PCR) in the lesioned tissues and in the blood of the clinically affected sheep and goats. Surprisingly, ORFV was also detected in the blood of healthy goats from an affected herd. Microscopically, they found a pseudo-papillomatous proliferation of the epithelium, while the dermis and lamina propria were expanded by a proliferating neovascular component that highly expressed the viral vascular endothelial growth factor (vVEGF) and its host receptor vascular endothelial growth factor receptor 2 (VEGFR2). Immunohistochemistry, immunofluorescence, and *in situ* hybridization for mRNA showed that epidermal growth factor receptor (EGFR) was expressed in the fibrovascular component, in the infiltrating CD163+ macrophages, and in the basal stratum of the epidermis. Confocal immunofluorescence microscopy demonstrated that CD163+ macrophages were associated with VEGF and VEGFR2. Finally, they found by quantitative RT-PCR the overexpression of the *interleukin-6* and *VEGFR2* genes in the lesioned tissues. These findings suggest that ORFV activates an inflammatory reaction characterized by CD163+ macrophages expressing EGFR and VEGFR2, which might play an oncogenic role through synergistic action with vVEGF signaling.

ORF virus (ORFV, family: *Poxviridae*) is a large double-stranded DNA virus belonging to the genus *Parapoxvirus* that causes contagious ecthyma (ORF), a disease affecting particularly sheep and goats, as well as humans, and a wide range of other host species which exhibit similar pathological changes.^1,2,7,9^ After infecting epidermal keratinocytes, ORFV triggers a sequence of typically self-resolving changes in the skin and mucosae. Lesions especially appear in the buccal fissure and in the oral cavity, beginning as erythema with vesicles and pustules, and sometimes forming severe papilloma-like lesions.^9^ The tumor-like changes during ORFV infection are considered atypical forms and are described in several species, including humans, in which they are indicated by different terms, namely “giant ORF lesions” and “pyogenic granuloma.”^12,26^ These tumor-like lesions should be unsurprising, given that one of the key pathological molecular factors encoded by ORFV is viral vascular endothelial growth factor (vVEGF), which represents the viral counterpart of the host’s intrinsic vascular endothelial growth factor (VEGF), driving the neovascular proliferation in skin and mucosae of the infected host.^23^ Similarly, in physiological and pathological conditions, such as wound healing and various neoplastic processes, the homologous mammalian protein of the viral VEGF protein, together with its receptors, vascular endothelial growth factor receptor 1 and 2 (VEGFR1 and VEGFR2), promotes neoangiogenesis and lymphangiogenesis by acting as a potent mitogen for endothelial cells.^8,9^

Indeed, one of the characteristic changes found in ORFV-affected skin is the presence of a marked neovascular component which is observed in all of the infected host species, including humans.^12,33^ Interestingly, in epidermal tumoral cells of K5-SOS transgenic mice, VEGF signaling synergizes with epidermal growth factor receptor (EGFR) to enhance the development of epithelial tumors, thus confirming its role in the genesis of various types of tumors.^21^

In humans and mouse models, numerous studies have demonstrated the role of EGFR in embryogenesis and in the development of several tumors, including head and neck tumors, glioblastomas, and colorectal carcinomas.^35^ In addition, EGFR expression by infiltrating activated macrophages, namely tumor-associated macrophages, has been described as essential for tumor growth in liver carcinoma and colorectal adenocarcinoma.^18,19^ Inflammation, with its molecular factors and cellular components, is an important environmental aspect of tumors.^24^ Complex relationships between tumors and inflammation exist; genetic alterations can activate oncogenes leading to neoplasia and inflammation, and chronic inflammatory conditions may increase cancer risk.^24^ In this regard, inflammation caused by bacterial and viral infections is considered a supporting event for developing tumors.^29,31^ It is well documented that certain viruses contribute to oncogenesis not only by promoting inflammation, which results in a protumoral change of the microenvironment, but even by expressing viral oncogenes.^28^ ORFV encodes several biochemical determinants, including a BCL-2-like factor with antiapoptotic properties, an interleukin-10-like cytokine, and various inhibitors of the nuclear factor-κB signaling pathway, which shape the inflammatory environment in host tissues.^9^ In addition, it has recently been found that ORFV encodes ORFV113 protein, which increases p38 phosphorylation by interacting with 1 of the 6 G-protein-coupled receptors (named LPA_1_) of lysophosphatidic acid, thus enhancing tumor progression and severity of the lesion.^16^ Interestingly, lysophosphatidic acid and its G-protein coupled receptors have multiple biological functions, including stimulation of cell proliferation and migration.^28^

Considering all these findings, we were prompted to study whether the inflammation occurring during natural ORFV infection in small ruminants may somehow trigger oncogenic processes. In particular, we focused on the roles of EGFR and macrophages as a consequence of the host inflammatory response to ORFV infection.

## Materials and Methods

### Ethical Statement

For diagnostic investigation purposes, samples were collected from ORFV-affected sheep and goats by using the procedure I 09 044 approved by the ethical committee of the Istituto Zooprofilattico Sperimentale (IZS) of Sardinia and regulated by the Italian Ministry of Health in the case of outbreak of viral infectious diseases. Bluetongue virus–infected samples that were used in the study as controls originated from a previous experimental infection study authorized by the Italian Ministry of Health (grant no. RC IZS SA 01/13). All testing described herein was performed at IZS, except electron microscopy (EM) that was performed at the Infectious Diseases Pathology Branch, Centers for Disease Control and Prevention, Atlanta, Georgia. Permission for the use of the samples was obtained from the owners.

### Animals and Sampling

Our study was performed in naturally ORFV-infected animals, including 15 lambs (*N* = 7 Sarda, *N* = 8 Lacaune) and 15 Saanen kids aged 10–20 days from 4 sheep and 3 goat farms with high incidences (10–30%) of ORFV infection, respectively. One 4-year-old ram from a flock without history of ORF was also included in our study. All animals selected for the study showed similar, severe ORFV lesions, characterized by an atypical tumor-like appearance. Animals with milder, classical lesions were not enrolled in the study. Ten milliliters of blood from every animal were collected in EDTA vacutainers, followed by humane euthanasia and pathologic examination with sampling of skin and mucosal lesions. Samples were split into 2 parts, one fixed in formalin and the other frozen at −80°C. ORFV was detected in all animals included in the study by real-time polymerase chain reaction (RT-PCR) and/or isolation in cell culture. Virus was isolated from the lesioned skin, buccal mucosae, and blood. Age-matched uninfected 9 sheep and 9 goats from two different farms without a history of ORFV infection were used as healthy controls.

In one ORFV-infected goat herd, a time-course study was performed on blood samples for detection of ORFV by RT-PCR and viral isolation, throughout clinical convalescence. Ten milliliters of blood were collected in EDTA from 3 goat kids at 7 time points (1, 7, 15, 35, 45, 55, and 65 days) after the clinical onset of disease. These kids were individually housed in an isolated pen throughout the course of sampling and clinical recovery. In addition, in the same herd, a single blood sample was taken from 105 healthy goats ≥2 years of age for ORFV detection by RT-PCR, to evaluate for subclinical infection.

### Histopathology

Formalin-fixed tissues were embedded in paraffin according to routine laboratory protocols, cut into 4-μm-thick sections, and stained using the ST Infinity Haematoxylin & Eosin Staining System (Leica Biosystems, Richmond, Illinois). Stained sections were examined under a light microscope with different magnifications, ranging from 50× to 400×.

### Immunohistochemistry (IHC) and Immunofluorescence (IF)

IHC and IF were performed on selected tissue sections for selected cluster of differentiation markers (CD immunophenotyping) of inflammatory cells and to characterize cellular receptors and protein expression in keratinocytes and mesenchymal cells in the skin and mucosa. To accomplish this, sections were deparaffinized and rehydrated using routine procedures and then, to expose antigen epitopes, heat-induced antigen retrieval was performed in Target Retrieval Solution pH 9.0 (Dako Agilent, Santa Clara, California) or citrate buffer pH 6.0 (Thermo Fisher Scientific, Waltham, Massachusetts). To quench endogenous peroxidase activity, slides were immersed in 0.3% H_2_O_2_ for 35 minutes at room temperature followed by 5% bovine serum albumin in phosphate-buffered saline to block nonspecific sites. Then sections were incubated overnight at 4°C using the primary antibodies as described in Table 1. We selected antibodies according to references and manufacturing company data.^25^ Moreover, validation was performed in our laboratory by demonstrating the expected tissue and cellular localization for each marker in goat and sheep tissues from this study.

**Table 1.** Details of immunohistochemistry and immunofluorescence antibodies.

Specificity	Ab Clone	Host	Source	Dilution	Antigen retrieval
Vimentin	V9	Mouse	Dako Agilent	1:100	Citrate buffer, pH 6.0 (pressure cooker 15 minutes at 110°C)
von Willebrand factor	Polyclonal	Rabbit	Dako Agilent	1:1300	EDTA buffer, pH 9.0 (pressure cooker 15 minutes at 110°C)
SMA	1A4	Mouse	Dako Agilent	1:50	Citrate buffer, pH 6.0 (pressure cooker 15 minutes at 110°C)
CD3	F7.2.38	Rabbit	Dako Agilent	1:1500	EDTA buffer, pH 9.0 (pressure cooker 15 minutes at 110°C)
CDαcy 79	HM57	Mouse	Dako Agilent	1:750	EDTA buffer, pH 9.0 (pressure cooker 15 minutes at 110°C)
CD68	514H12	Mouse	Bio-Rad	1:100	EDTA buffer, pH 9.0 (pressure cooker 15 minutes at 110°C)
CD163	EDHu-1	Mouse	Bio-Rad	1:800	Citrate buffer, pH 6.0 (pressure cooker 15 minutes at 110°C)
VEGF	Polyclonal	Rabbit	Millipore	1:1000	Citrate buffer, pH 6.0 (pressure cooker 15 minutes at 110°C)
VEGFR1	D-2	Mouse	Santa Cruz	1:50	EDTA buffer, pH 9.0 (pressure cooker 15 minutes at 110°C)
VEGFR2	D-8	Mouse	Santa Cruz	1:30	Citrate buffer, pH 6.0 (pressure cooker 15 minutes at 110°C)
VEGFR2^a^	SP-123	Rabbit	Abcam	1:150	Citrate buffer, pH 6.0 (pressure cooker 15 minutes at 110°C)
EGFR	EP38.Y	Rabbit	Abcam	1:75	Citrate buffer, pH 6.0 (pressure cooker 15 minutes at 110°C)

Abbreviations: Ab, antibody; SMA, smooth muscle actin; VEGF, vascular endothelial growth factor; VEGFR1, vascular endothelial growth factor receptor 1; VEGFR2, vascular endothelial growth factor receptor 2; EGFR, epidermal growth factor receptor.

a This antibody was preferred in multicolor immunofluorescence studies in accordance with other antibodies and the species in which they were raised

The anti-VEGF antibody was tested in humans but was previously shown to cross-react with the ovine VEGF protein; therefore, based on the homology, we expected binding with vVEGF.^32^ IHC was carried out using a Dako EnVision kit (Dako Agilent, Santa Clara, California); this was followed by a 5-minute incubation with 3,3′-diaminobenzidine substrate-chromogen (Dako Agilent, Santa Clara, California) and counterstaining with Mayer’s hematoxylin.

IF studies were carried out using the same repertoire of primary antibodies, at the same dilution and antigen retrieval treatment, except for VEGFR2 (Table 1). Secondary antibodies conjugated with fluorescent dyes were used for detection (Alexa Fluor 488 and Alexa Fluor 599; Thermo Fisher Scientific, Waltham, Massachusetts), and ProLong Gold Antifade Mountant with DAPI (Thermo Fisher Scientific, Waltham, Massachusetts) was used to mount coverslips on the slides.

Each IHC and IF assay included appropriate negative controls run in parallel. Negative controls for ORFV IHC and IF assays included lip, gum, tongue, and axilla skin sections of healthy lambs and kids that were negative by RT-PCR for ORFV. Negative controls were also performed by replacing the primary antibody with nonimmune serum. Nonlesional areas, included in the same slide, from ORFV-affected animal tissues were used as internal controls for some of these markers, considering the expected tissue and cellular localization. Positive controls for CD markers included lymph-node and tonsil sections from uninfected small ruminants.

In the IF run, EGFR expression during non-ORFV inflammation was estimated in axilla skin, tongue, and lip samples from 10 sheep experimentally infected, in previous studies, with bluetongue virus (serotype 1 and 8) and euthanized 5 or 10 days after. In these sheep, inflammatory patterns were characterized by mononuclear inflammatory cells in the dermis and lamina propria. Leica DM 4000 B and Leica TCS-SP8 confocal microscopes were used to capture images.

### Electron Microscopy

Ultrastructural examination was performed on 4-µm-thick formalin-fixed paraffin-embedded sections of *Ovis aries* gum affixed to glass slides (“on-slide”). Areas of interest for EM from on-slide sections were selected based on corresponding areas showing positivity by ORFV *in situ* hybridization. On-slide sections were deparaffinized in xylene and rehydrated before being embedded in epoxy resin. Sections were then removed from glass slides by immersing the sections in boiling water and using a razor blade to separate the epoxy embedded section from the slide. Areas of interest were cut out and glued onto a blank EM block prior to thin sectioning. EM sections were stained with uranyl acetate and lead citrate and examined on a Tecnai Spirit electron microscope.

### *In Situ* Hybridization

For *in situ* hybridization, the detection of ORFV mRNA and *EGFR mRNA* was performed by using RNA-scope 2.5 Red assay kit (cat 322360; Advanced Cell Diagnostics [ACD], Hayward, California). The RNA-scope probes were designed to detect the mRNA expression of the ORFV *BL2* gene and *EGFR*. Deparaffinized and rehydrated 5-µm-thick tissue sections were incubated in H_2_O_2_ for 10 minutes followed by target retrieval buffer (ACD, Hayward, California) at 98–100°C using a hot plate for 15 minutes. Tissue sections were then treated with Protease Plus reagent at 40°C for 25 minutes in a hybridization oven (ACD, Hayward, California) and subsequently incubated at 40°C with the target probe in the same hybridization oven for 2 hours. We used probes targeting the *PPIB* (*Cyclophilin B*) and *DapB* (Bacillus subtitlis strain SMY) genes, as positive and negative controls, respectively.

The mRNA expression of *interleukin-6* (*IL-6*) was detected with Invitrogen ViewRNA ISH Tissue Assay Core Kit (Cat. No. 19931; Invitrogen, Waltham, Massachusetts) as per the manufacturer’s recommendations and using the ViewRNA Probe (Cat. No. VF1-4261131, Invitrogen, Waltham, Massachusetts). Deparaffinized and dehydrated tissue sections were incubated at 90–95°C in a pretreatment solution on a hot plate for 10 minutes. Sections were then submitted to protease (Cat. No. VF1-4261131, Invitrogen, Waltham, Massachusetts) digestion for 10 minutes in a hybridization oven (Dako hybridizer Stat Spin) and incubated at 40°C in the same oven for 2 hours. The positive control was a probe targeting the *Ovis aries beta actin* gene. As a negative control, the target probe set was omitted. A series of target-specific signal-amplification steps were done for both assay kits. FAST Red signal-amplification systems (ACD and Invitrogen, respectively) were then hybridized to the target probes. Slides were counterstained with hematoxylin and cover slipped using Ecomount (Biocare medical, Concord, California). Sections were examined under a light microscope (Leica DM4000B).

### Virology

Virus isolation was performed by passing homogenates of injured tissues and sonicated blood on semiconfluent monolayers of 154th passage Vero cells (African green monkey BSCL86 ATCC, IZS of Lombardy and Emilia Romagna, Italy) at 37°C and 5% CO_2_. The cultures were checked daily for the presence of cytopathic effect under a light-inverted microscope (Olympus mod. IX73) with 20× and 40× magnifications. Cytopathic effect, which included rounding and ballooning, was detected approximately 2 days after infection. When 80% cytopathic effect was observed, cells and media were frozen at −80°C then thawed and centrifuged at 800 ×*g* for 15 minutes at 4°C; the supernatants were collected and stored at −80°C. RT-PCR techniques were used to confirm presence of parapoxvirus, as previously described.^10^

A second set of blood and tissues from the same lesions used for virus isolation was used for molecular analysis. Total DNA was extracted from 25 mg of tissue or 200 µL of blood using the Dneasy Blood and Tissue Kit (Qiagen, Hilden) according to the manufacturer’s instructions. ORFV DNA in blood and tissues was detected by RT-PCR as described by others.^10^

### RT Gene Expression Quantification

In the lips of affected kids (*N* = 15) and lambs (*N* = 15), and healthy lamb controls (*N* = 18), *IL-6* (Thermo Fisher custom assay ID APCE36N), *VEGF* (Thermo Fisher assay ID Oa0453812_m1), *VEGFR2* (Thermo Fisher assay ID Oa046 56481_m1), *VEGFR1* (Thermo Fisher assay ID Oa0439270_m1), and *EGFR* (Thermo Fisher assay ID Oa04798940_g1) mRNA and *18s* rRNA (housekeeping gene) were isolated by using RNeasy Fibrous Tissues mini kit (Qiagen, Hilden), and then treated with DNAse. After that, RNAs were amplified by quantitative real-time PCR (qRT-PCR) from 50 ng of polyA RNA purified from the total ORFV-affected tissue RNA using the Oligotex mRNA kit (Qiagen, Hilden).

Various housekeeping genes were tested, including *18s*, *beta-actin*, and *hemoglobin beta 2. 18s* and *beta-actin* results were comparable, and therefore both were used in each run.

Probe sequences for the host *VEGF* expression quantification were designed to be a perfect match of the ovine and caprine mRNA, thus avoiding any misestimation due to the presence of the *vVEGF* mRNA. Reverse transcription was performed by using a Quantitect Reverse Transcription Kit (Qiagen, Hilden). Data obtained were analyzed with the Sequence Detection Systems 2.3 software (Life Technologies, Waltham, Massachusetts) and the relative *IL-6*, *VEGF*, *VEGFR2*, *VEGFR1*, and *EGFR* gene expression levels were calculated using the 2^-ΔΔCT^ method.

### Statistical Analysis

Normality distributions of the collected variables (*IL-6*, *VEGF*, *VEGFR2*, *VEGFR1*, and *EGFR*) were assessed with the Shapiro-Wilk normality test. Median and interquartile range were used to describe quantitative variables. Statistical differences of gene expression between healthy and affected animals under study were evaluated by performing the Mann-Whitney test, and a two-tailed *P*-value <.05 was considered statistically significant. Analysis was performed with Stata (StataCorp, College Station, Texas).

## Results

### Clinical and Pathology Findings and ORFV Localization in Tissue

**Figure 1.**
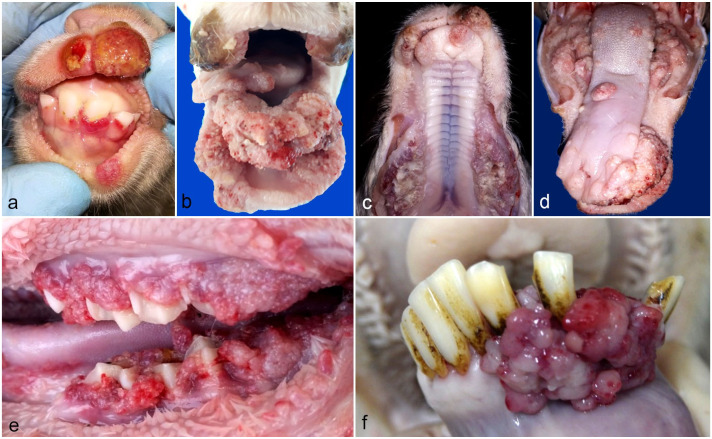
ORF virus infected lambs, kids, and ram. Representative gross and microscopic findings in the buccal mucosae and skin. **(a)** Well-circumscribed red masses in the gingiva and lips of a lamb. **(b, c)** Coalescing hyperemic firm nodules with cauliflower-like appearance covering the line of the incisive and molar teeth in a kid and lamb, respectively. **(d, e)** Single and coalescing nodules in the browsing pad, margins of the lips, palate, and tongue in a kid. **(f)** Isolated multilobular hyperemic mass arising among the incisors tooth of an adult ram.

Lambs and kids became clinically affected by ORFV at 3–15 days of age. The younger animals displayed earlier and milder lesions appearing as bright red, 0.5–1 cm masses in the gingival mucosa surrounding the corona of the incisor teeth when they were erupting, with papules, which were occasionally ulcerated, present in the lip skin (Fig. 1a). In the severe atypical cases, lesions appeared as multiple coalescing, hyperemic, firm nodules with cauliflower-like surfaces in the gingiva, dental pad, margins of the lips, palate, and tongue (Fig. 1b–e). These hyperplastic formations protruded 2–3 cm above the mucosal surface, extending over the incisor and molar teeth in the more severe cases. Similar but crusted and isolated growths were rarely observed in the coronet of the hoof or in the skin of the tail. Interestingly, during our survey, in one adult ram belonging to a flock with no history of ORFV infection, we found a single 3 cm × 2 cm irregular, hyperemic, slowly growing, multinodular mass in the gingiva between the incisor teeth (Fig. 1f).

**Figure 2.**
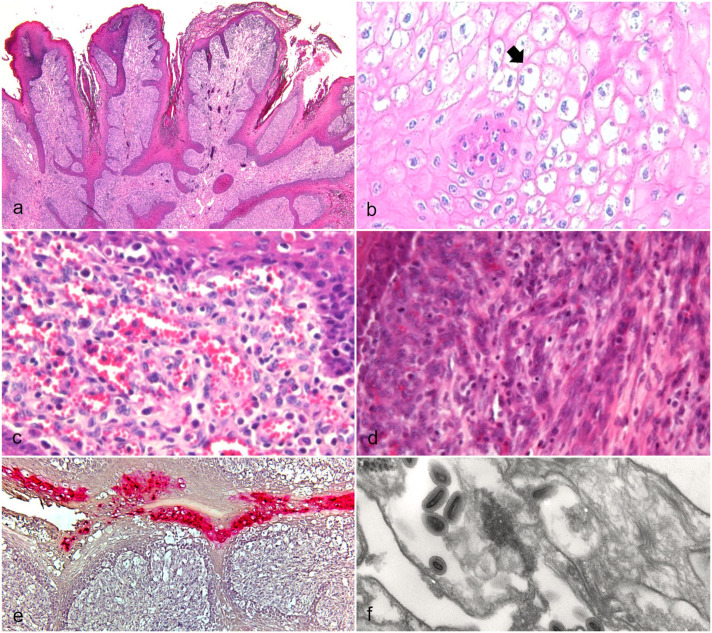
ORFV infected lambs and kids. ORFV, ORF virus. **(a)** Tumor-like proliferation of the gingival epithelium characterized by hyperkeratosis and elongated rete ridges sustained by fibrovascular structure. Lamb. Hematoxylin and eosin (HE). (**b)** Keratinocytes with hydropic alterations, formation of koilocytes, and presence of cytoplasmic eosinophilic inclusion bodies (arrow) in the skin of an affected lamb. HE. **(c)** A representative area of newly formed vessels in the lamina propria. Kid. HE. **(d)** A different area, from the same case shown in (c), displayed densely packed fibrovascular cells with no new organized vessels. HE. **(e)** mRNA localization (red) of ORFV is found only in the damaged keratinocytes. Kid. RNA-scope *in situ* hybridization for ORFV mRNA. **(f)** Electron micrograph of lesioned mucosa of a lamb shows parapoxvirus particles.

Among these different types of macroscopic lesions, we focused our detailed pathological investigation on the severe atypical lesions that lacked ulceration or suspected secondary bacterial infection. Lesions evaluated were from 15 lambs, 15 kids, and 1 adult ram. The histopathological findings and results of IHC and in situ hybridization assays did not differ qualitatively between sheep and goat specimens, or between cutaneous and mucosal lesions. By histological examination, we found markedly pseudo-papillomatous proliferations of the epithelium with ortho- and parakeratotic hyperkeratosis and elongated rete ridges (Fig. 2a). Keratinocytes displayed hydropic alterations and edema, with koilocyte formation. Increased keratohyalin granules and rare eosinophilic, intracytoplasmic inclusion bodies were also observed (Fig. 2b).

The dermis and the lamina propria were expanded by proliferating mesenchymal cells that had indiscrete cytoplasm and irregularly rounded or elongated nuclei that were densely packed in linear arrays or divided by gaps representing a network of newly formed vascular spaces containing erythrocytes (Fig. 2c, d). These neovascular formations were notable, with their histological density and undifferentiated status correlating with the severity of the proliferative aspect of the gross lesion. By *in situ* hybridization and EM, parapox viral mRNA and viral particles, respectively, were detected exclusively in the epithelium of the mucosal and skin lesions (Fig. 2e, f).

### VEGF and EGFR Expression in Atypical Tumor-Like ORF Epithelial and Vascular Proliferative Lesions

**Figure 3.**
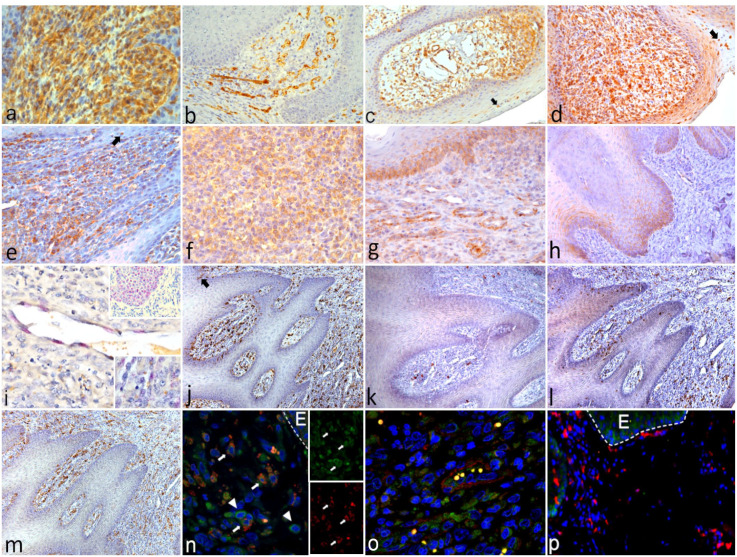
Representative photomicrographs of the immunohistochemistry (IHC) and immunofluorescene findings. **(a–d)** ORF virus infected kid. Buccal mucosae. **(a)** Proliferating fibrovascular component in the lamina propria showed diffuse immunolabeling for vimentin. Vimentin IHC. **(b)** Immunoreactivity for von Willebrand factor (vWF) outlines the newly formed vessels. vWF IHC. **(c)** Abundant immunolabeling for vascular endothelial growth factor receptor (2) (VEGFR2) of fibrovascular component in the lamina propria, which contains morphologically distinct vessels. Macrophages (arrow) infiltrating the epithelium show immunohistochemical reactivity for VEGFR2. VEGFR2 IHC. **(d)** Immunolabeling of vascular endothelial growth factor (VEGF) is observed in the lamina propria and associated with macrophages (arrow) infiltrating the epithelium. VEGF IHC. **(e–g)** ORF virus infected lamb. Buccal mucosa. **(e)** Representative area of the mucosa displaying epidermal growth factor receptor (EGFR) immunolabeling in the lamina propria. EGFR labeling in the epithelium is also associated with large macrophages (arrow). EGFR IHC. **(f)** Representative area of the lamina propria showing densely packed cells with EGFR immunolabeling. EGFR IHC. **(g)** Newly formed vessels showing immunolabeling for EGFR. EGFR IHC. **(h)** Healthy control lamb, buccal mucosa. Moderate immunolabeling for EGFR in the basal stratum of the epithelium. **(i)** *EGFR* mRNA localization in the endothelial cells of the newly formed vessels, in the epithelium (inset at the top right corner) and, with distinct spots patterns, in the fibrovascular component (inset at the bottom right corner). RNA-scope *in situ* hybridization for *EGFR* mRNA. **(j–m)** Serial sections of the same area of the buccal mucosa of ORF virus-infected lamb. **(j)** Diffuse immunolabeling of CD163+ macrophages infiltrating the mucosa. Few macrophages also infiltrate the epithelium (arrow). CD163 IHC. **(k)** Immunolabeling of few CD68+ macrophages. CD68 IHC. **(l)** IHC for CD3+ T lymphocytes. CD3 IHC. **(m)** IHC for CD79αcy+ B lymphocytes. CD79αcy IHC. **(n–p)** Immunofluorescence and confocal microscopy of the buccal mucosa of an ORFV-infected lamb. **(n)** In the merged image, EGFR is expressed on CD163+ macrophages (arrow) and other cells of the fibrovascular component (arrowhead). The 2 insets on the right represent the single separate images of EGFR (in green) and CD163 marker (in red). DAPI nuclear stain in blue. E, epithelium. **(o** In a serial section of Fig. 3n, SMA + pericytes surround endothelial cells expressing EGFR in a newly formed vessel containing erythrocytes. SMA, smooth muscle actin. SMA is shown in red, EGFR in green, and DAPI nuclear stain in blue. **(p)** Immunofluorescence and confocal microscopy of the lamina propria of the lip mucosa from a bluetongue virus sheep. No expression of EGFR was observed in the lamina propria which displays numerous CD163+ macrophages, while the receptor is observed in the basal layer of the epithelium. EGFR is shown in green, CD163 in red, and DAPI nuclear stain in blue. E, epithelium.

In both species, to characterize the fibrovascular component observed in the dermis and lamina propria, we used antibodies against vimentin and various endothelial cell markers, including von Willebrand factor, VEGFR2, VEGFR1, and VEGF. We found that in the dermis and the lamina propria the spindle cells intensely immunolabeled for vimentin, as well as VEGFR2 and von Willebrand factor, which were also detected in newly formed blood vessels (Fig. 3a–c). VEGF immunolabeling patterns were similar to those observed for its receptor VEGFR2 in the dermis and lamina propria (Fig. 3d). Notably, VEGFR2 and VEGF immunoreactivity was also observed in the epithelium associated with the basal stratum and to single infiltrating cells resembling macrophages (Fig. 3c, d). On the contrary, the immunohistochemical expression of VEGFR1 was very weak and relegated to few subepithelial areas.

**Figure 4.**
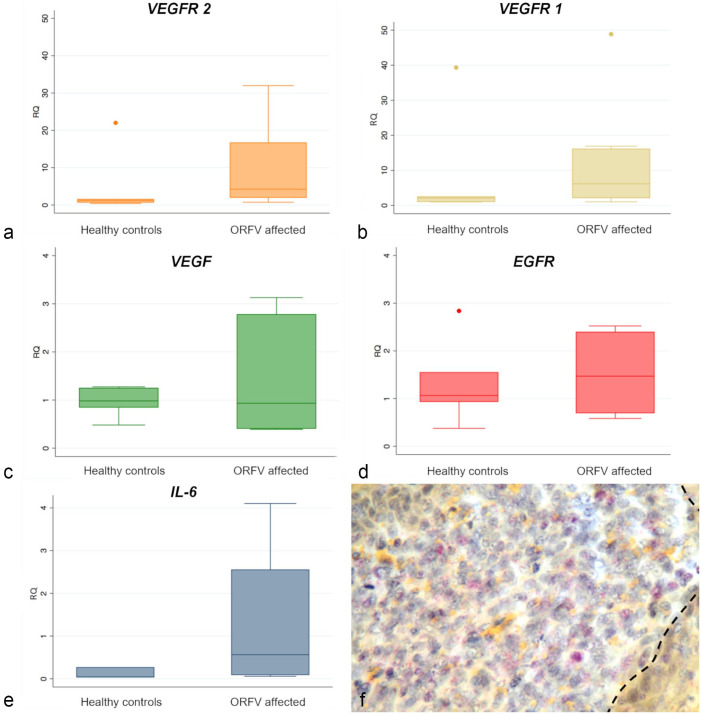
Graphs and photomicrograph representing the relative levels of gene expression by quantitative real-time PCR in ORF virus infected and healthy control lambs and kids. **(a–e)**. Gene expression relative quantification of host *vascular endothelial growth factor receptor 2* (*VEGFR2*), *vascular endothelial growth factor receptor 1* (*VEGFR1*), *vascular endothelial growth factor* (*VEGF*), *epidermal growth factor receptor* (*EGFR*), and *interleukin-6* (*IL-6*). Statistically significant overexpression was found only for *VEGFR1*, *VEGFR2*, and *IL-6* when compared with the healthy controls. Values were normalized to the expression levels of the sheep *18s* rRNA and *β-actin* (*ACTB*) and expressed as relative fold change compared to the expression levels of one of the healthy controls. **(f)** Representative image of the *IL-6* mRNA localization in the dermis. In situ hybridization for *IL-6* mRNA.

*VEGFR2* and *VEGFR1* gene expression (*P* ≤ .002 and .03, respectively), as assessed by qRT-PCR, was significantly higher in the ORFV-affected tissues when compared to those from the healthy controls (Fig. 4a, b), while no differences in expression were found for host VEGF and EGFR (Fig. 4c, d).

Since VEGF has previously been reported to synergize with EGFR in promoting tumor development, we used IHC, qRT-PCR, and confocal microscopy to determine whether EGFR was overexpressed in the ORFV-infected tissues. We found that EGFR was immunohistochemically expressed in the dermis/lamina propria and basal stratum of the epithelium of the skin and mucosa (Fig. 3e–g). In addition, endothelial cells of the newly formed vessels also showed immunoreactivity for EGFR (Fig. 3g). Interestingly, intraepidermal macrophages also immunolabeled for EGFR and CD163 (Fig. 3e, j). In the uninfected control, EGFR was weakly expressed in the basal stratum of the epithelium (Fig. 3h). To further confirm the activity of EGFR in ORFV-infected tissues, we determined its mRNA *in situ* expression with RNA-scope ISH. Mirroring the immunolabeling patterns of the protein, the expression of *EGFR* mRNA was observed in the basal stratum of the epithelium, in the endothelium of vessels, and in several cells of the dermis/lamina propria (Fig. 3i). As expected, a very low *EGFR* mRNA expression was also detected in the uninfected control, in the basal stratum of the epithelium. By qRT-PCR, we found no relative difference of *EGFR* gene expression between ORFV-infected tissues and healthy samples (Fig. 4d).

### CD163+ Macrophages Express EGFR and VEGFR2 in Atypical ORF Lesions

To better understand the host immune response to ORFV infection, the inflammatory infiltrate associated with the proliferative lesions of ORFV was characterized by IHC. We used monoclonal antibodies against CD163 and CD68 to identify macrophages, and CD3 and CD79αcy for T lymphocytes and B lymphocytes, respectively.

In lambs and kids, the immunohistochemical results demonstrated a mixture of inflammatory cells that included macrophages and lymphocytes in the dermis, the lamina propria, and the epidermis of affected animals. Importantly, we found that CD163+ macrophages were by far the most represented inflammatory cells (Fig. 3j–m), with some CD163+ macrophages infiltrating the epithelium (Fig. 3j). Few CD68+ macrophages were observed throughout the inflamed areas of the dermis and lamina propria. Confocal microscopy results demonstrated that EGFR expression was associated with numerous CD163+ macrophages situated in the dermis/lamina propria or infiltrating the epithelium, and that this receptor was also expressed by endothelial cells (Fig. 3n, o).

To investigate whether ovine CD163+ macrophages expressed EGFR under other physiological or pathological conditions, we used IF to analyze the tongue mucosa from bluetongue virus sheep with severe inflammation in the mucosae and skin. Results showed that the numerous CD163+ macrophages infiltrating the lamina propria and the dermis of bluetongue virus sheep were negative for EGFR (Fig. 3p).

**Figure 5.**
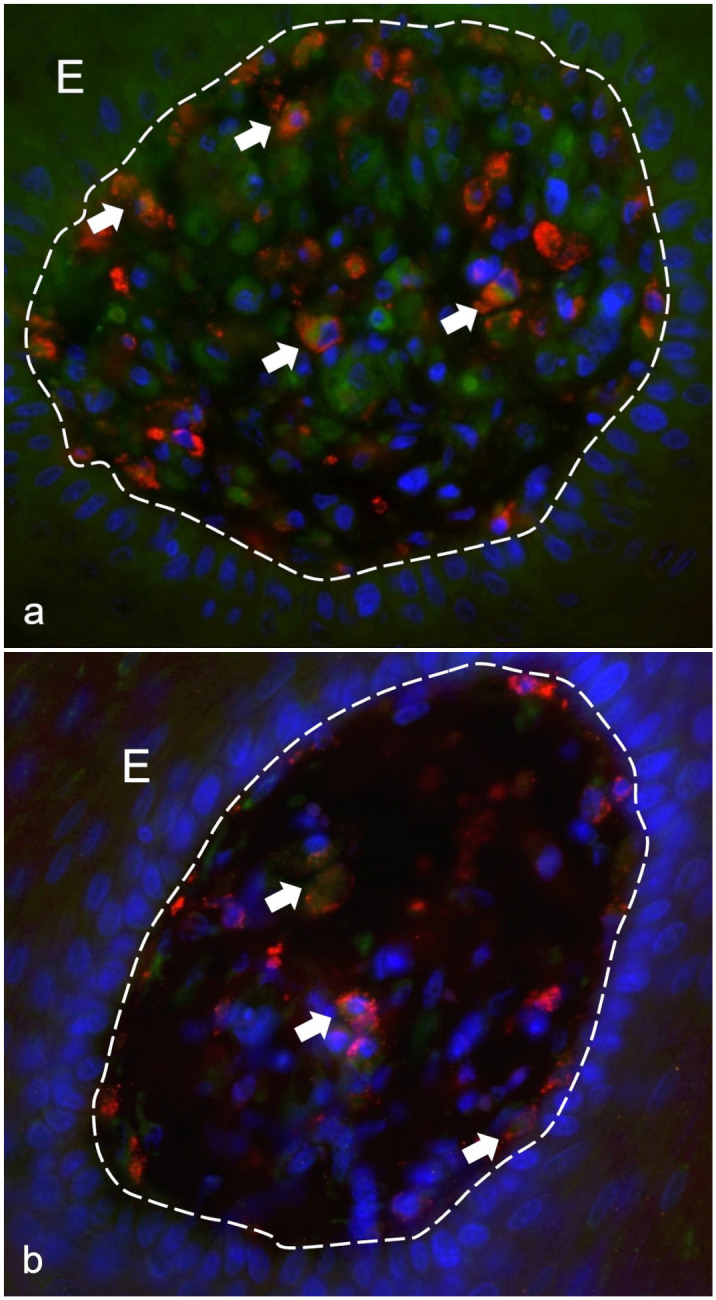
Confocal microscopy of the lamina propria of the mucosa from an ORF virus infected kid. (a) Vascular endothelial growth factor receptor 2 (VEGFR2) is expressed in CD163+ macrophages (arrows). VEGFR2 is shown in green, CD163+ macrophages in red, and DAPI nuclear stain in blue. (b) Vascular endothelial growth factor (VEGF) immunolabeling is present in CD163+ macrophages (arrows). VEGF is shown in green, CD163+ macrophages in red, and DAPI nuclear stain in blue. E, epithelium.

It has been reported that macrophages can express VEGFR2 in a tumor microenvironment.^6^ By using double IF, we confirmed that CD163+ macrophages infiltrating the dermis/lamina propria and the epithelium of the ORFV-infected tissues also coexpressed VEGFR2 (Fig. 5a). Thus, because of their VEGFR2 expression, CD163+ macrophages are suspected to also bind vVEGF (Fig. 5b). No differences were observed between lambs and goats.

As it is well known that IL-6 is critical in shaping tumor-associated inflammation and in promoting cell proliferation after being produced by EGFR-expressing macrophages, we decided to evaluate gene expression by qRT-PCR of this proinflammatory cytokine. Results demonstrated statistically significant higher (*P* ≤ .015) expression of *IL-6* in ORFV-infected tissues compared to uninfected controls (Fig. 4e). *In situ* hybridization of *IL-6* was mostly found in the dermis/lamina propria of the ORFV-infected tissues (Fig. 4f).

### Persistent and Latent ORFV Infections Occur in Clinically Healthy Goats

The time-course study in the clinically affected kids (*N* = 3) yielded detection of ORFV by RT-PCR from the blood until the last testing day at 65 days after lesion onset (Table 2). At this point, all the 3 animals had recovered from the clinical disease. The presence of ORFV in the blood was also confirmed by isolation in cell culture at the first testing day (Table 2). Interestingly, ORFV DNA was also detected by RT-PCR in the blood from 64.5% to 83.9% healthy goats belonging to an affected farm (Table 3).

**Table 2.** Detection of ORF virus by RT-PCR and cell culture in the blood of affected kids after the clinical onset of the disease.

	Days Postclinical Onset							
	1	1	7	15	35	45	55	65
No. of Kid	Cell Culture	RT-PCR Ct Values						
1	+	27.5	27.6	29	32	35	37.1	37.9^a^
2	+	30.1	32.4	33.2	34	34.5	34.3	34.9^a^
3	+	28.4	28.9	31.2	32.7	32.3	34.5	34.5

Threshold cycle (Ct) values of <40 are considered positive.

Abbreviations:+, positive; RT-PCR, real-time polymerase chain reaction.

a Recovery.

**Table 3.** Prevalence of clinically healthy goats at different ages with ORF virus-positive blood by RT-PCR from an affected herd displaying numerous clinical cases.

Range of Age (Years)	Prevalence % (Positive/Examined)	Range of RT-PCR Ct Values
1–2	84 (26/31)	27.3–36.1
2–4	67 (29/43)	30.1–38.3
4–7	65 (20/31)	29–36.5

Threshold cycle (Ct) values of <40 are considered positive.

Abbreviation: RT-PCR, real-time polymerase chain reaction.

## Discussion

Upon histological examination of natural ORF cases included in this study, we have been impressed by the prominent, atypical tumor-like aspects of the lesions, including papillomatous epithelial hyperplasia and angiomatous vascular proliferation, which have been previously described in animals and in humans.^9,34^ The morphological and immunohistochemical findings in our study indicated that the proliferating tissue in the dermis and lamina propria of these lesions was comprised of a fibrovascular component undergoing angiogenesis.

These findings prompted us to investigate the role of well-known tumor promoting signaling pathways in the genesis of the atypical proliferative lesions during ORFV infection in sheep and goats. Experimental infection of sheep with a recombinant ORFV strain has previously shown that the severity of epithelial hyperplasia and neovascularization correlates with the presence of the active *vVEGF* gene.^33^ We found that ORFV activates inflammation characterized by CD163+ macrophages, and increases host EGFR expression and *IL-6* production, which might promote oncogenesis through synergistic action with vVEGF signaling.

We demonstrated the expression of vVEGF by IHC and extensive expression of its specific receptor, VEGFR2 by IHC and qRT-PCR. Together with the sparse IHC staining for VEGFR1, which binds host VEGF, this suggests that VEGF immunohistochemical detection originated mostly from VEGF protein synthesized by the virus (ie, vVEGF). Our finding is consistent with the previous results indicating the significance of vVEGF in the pathogenesis of atypical ORF lesions.^27^ Although a significant expression of *VEGFR1* was also found by qRT-PCR, the lack of the host VEGF overexpression suggests that host VEGF does not play a major role in the angiogenesis observed. Further, we suspected that other factors besides vVEGF could be involved in the genesis of the proliferative ORFV lesions, because of the reported presence of minimally productive angiomatous changes in animals experimentally infected with ORFV lacking the *VEGF* gene.^33^

Investigating a potential role of EGFR, we found more intense EGFR labeling by IHC in the lesioned skin and mucosa of ORF-affected goats and sheep compared to uninfected controls. However, the 2^−ΔΔC^_T_ (relative) qRT-PCR did not show a difference of gene expression compared to controls. These data indicate an apparent lack of correlation between EGFR mRNA quantification and immunolabeling of the corresponding protein in the examined ORFV-affected skin and mucosa. This apparent mismatch is not surprising, given that no correlation between EGFR immunohistochemical expression and gene copy number, identified by fluorescence *in situ* hybridization, has been observed previously in human hepatocellular carcinoma.^3^ Similarly, comparative studies investigating EGFR expression in breast and colorectal cancer have found that *EGFR* gene amplification by qRT-PCR and EGFR protein expression by IHC are unrelated.^15,20^ Nevertheless, IHC is currently considered the most appropriate way to evaluate the expression of the EGFR protein in tumors.^5^ We speculate that the mismatch between EGFR gene expression and antigen expression is due to cells expressing protein in excess of the RNA copy number for EGFR, and to the longer stability and recycling of protein after activation.

By confocal IF and ISH, EGFR overexpression was localized in our cases to not only epithelial cells, as we expected, but also to endothelial cells and infiltrating CD163+ macrophages in the dermis and the lamina propria in tumor-like ORF lesions. Interestingly, in transgenic K5-SOS mouse, which develop spontaneous skin papillomas at 100% penetrance, it was demonstrated that depletion of *EGFR* expression in the mouse entirely prevents tumor formation, due to EGFR inhibition of tumoral cell apoptosis.^21^ Together, these findings support a synergistic effect of EGFR and VEGF signaling in papilloma development.^21^

These data suggest that EGFR is a molecular component which plays a role in the pathogenesis of the angiomatous and papillomatous aspects of the changes caused by ORFV, probably acting in synergy with VEGF. It is notable that VEGF appears to be able to act as growth factor for not only vascular endothelial cells but also epithelial cells in mouse models.^21^ Moreover, the absence of EGFR-expressing CD163+ macrophages in the tongue mucosa of bluetongue virus-infected or healthy sheep clearly indicates that the expression of this receptor is not the result of generic viral infection but may be unique to ORFV infection.

In our study, the immunohistochemical characterization of the inflammatory cells within proliferative lesions demonstrated the presence of predominantly CD163+ macrophages. The high expression of CD163 indicates that ORFV promotes an M2 phenotype in macrophages, which are considered tumor-promoting.^30^ In this context, it was demonstrated in transgenic mice that CD163+ macrophages expressing EGFR are essential in hepatocarcinogenesis, and their presence has powerful prognostic value.^18^ Similarly, the severity of activated tumor-promoting macrophage infiltration in colitis-associated carcinogenesis is modulated by EGFR signaling, such that myeloid EGFR knockout mice exhibit decreased tumoral growth associated with impaired angiogenesis.^13^

Another aspect we considered relevant is the expression of VEGFR2 in infiltrating CD163+ macrophages. Interestingly, the expression of this receptor is known to modulate macrophage infiltration in orthotopic pancreatic tumors in mice.^6^ In our cases, the same CD163+ macrophages immunolabeled for VEGF. We suspect that, in this case, VEGF is of viral origin and binds to CD163+ macrophages because of their expression of its receptor VEGFR2. The consequences of this signaling pathway in macrophages require further study. However, it should be noted that, in a mouse model of breast cancer caused by expression of the polyoma middle T oncoprotein, macrophages produce VEGF, which induces neoangiogenesis, leading to malignancy in mammary neoplasia.^22^ Similar processes may occur in ORF.

In our cases, ORFV-lesioned tissues significantly overexpressed *IL-6*, which is a multifunctional cytokine implicated in many proinflammatory functions, but also in promoting cell survival, apoptosis, and proliferation, with the level of IL-6 significantly increased in several tumors.^11,14,17^ Accordingly, EGFR activates transcription of *IL-6* in liver macrophages contributing to hepatocellular carcinoma development.^18^ These findings suggest that this cytokine is involved in the molecular mechanism leading to the atypical proliferative changes seen in some ORFV infections.

Finally, by cell culture and RT-PCR, we found ORFV in the blood of clinically affected and healthy, asymptomatic exposed goats, as recently reported in Chinese goats.^4^ Recovered animals had detection by RT-PCR to 65 days postclinical onset. These findings suggest that ORFV in infected herds displays both persistence and clinical latency in some goats. They also challenge the generally accepted notion that skin must be damaged for ORFV infection to occur. We speculate that immune status plays a determinant role in the outcomes of ORFV viremia in asymptomatic animals, particularly during the development of atypical productive lesions as observed in giant ORF lesions in man.^26^ Further studies are needed to evaluate whether ORFV is replicating in the blood, with what cells it is associated, and how long it persists in the blood of clinically healthy animals. Collectively, our morphological, virological, immunohistochemical, and gene expression findings strongly suggest that CD163+ macrophages and their EGFR expression have a fundamental role, through synergistic action with vVEGF signaling and the production of IL-6, in the development of the tumor-like productive lesions during ORFV infection. While further *in vitro* and *in vivo* experiments are needed to characterize the potential oncogenic effects of ORFV-induced inflammation, the findings reported herein suggest that atypical tumor-like ORF lesions in sheep and goats may provide a model in which to study viral oncogenic mechanisms.
